# The Association Between Transformational Leadership and Nurses' Clinical Performance in the Tabuk Health Cluster, Saudi Arabia

**DOI:** 10.1002/nop2.70708

**Published:** 2026-08-06

**Authors:** Ali Amer Albargi, Faroq Abdulghani Alshameri, Waled A. M. Ahmed, Mugahed Ali Alkhadher, Yahya Hussein Ahmed Abdalla, Abdualrahman Akasha Abdualrahman, Khaled Saleh Mohammed Algaradi, Fahad A. Alghamdi

**Affiliations:** ^1^ Faculty of Nursing Tabuk University Tabuk Saudi Arabia; ^2^ Medical and Surgical Nursing Department, Faculty of Nursing Tabuk University Tabuk Saudi Arabia; ^3^ Community Health Nursing Department, Faculty of Nursing Al‐Baha University Al‐Baha Saudi Arabia; ^4^ Medical and Surgical Nursing Department, Nursing College Najran University Najran Saudi Arabia; ^5^ Community Health Nursing. Community and Mental Health Department. Nursing College Najran University Najran Saudi Arabia; ^6^ Medical and Surgical Nursing Department Al‐Rayan National College of Nursing, Al‐Rayan National Colleges Al Madinah Al Munawwarah Saudi Arabia; ^7^ Al Liath General Hospital, The First Health Cluster in Jeddah Ministry of Health Jeddah Saudi Arabia; ^8^ Faculty of Nursing Al‐Baha University Al‐Baha Saudi Arabia

## Abstract

**Aim:**

This study examined the relationship between transformational leadership and clinical work performance among nursing professionals within the Tabuk Health Cluster. It focused specifically on the association of leadership behaviours with nursing performance.

**Design:**

The study utilized a cross‐sectional analytical quantitative research design.

**Methods:**

Participants: A convenience sample of 280 nursing professionals (staff nurses and head nurses) from within the Tabuk Health Cluster was enrolled. Measures: Transformational leadership was measured with the Global Transformational Leadership (GTL) scale, and clinical performance through a 51‐item scale derived from Yuxiu et al. (2011). Data Collection: This was a self‐administered online questionnaire conducted over approximately 6 weeks, between November and January 2024. Analysis: Data were analysed using SPSS version 26 with descriptive statistics, independent samples *t*‐tests, ANOVA, and multiple linear regression to determine the predictive value of transformational leadership.

**Results:**

Nurses perceived leadership positively (mean = 3.96/5). Overall clinical work performance was moderate (Mean = 2.70/4), with the leadership (2.75) and critical‐care (2.79) subscales scoring highest. Transformational leadership significantly predicted clinical performance (*p* < 0.001), accounting for 26.9% of its variance. Nurses with 1–5 years of critical‐care experience scored significantly higher than those with more than 5 years on both leadership perception and clinical performance (*p* < 0.001).

**Conclusion:**

Transformational leadership was positively associated with nurses' clinical performance and represents a modifiable target for improvement. The lower scores among more experienced nurses indicated a need to re‐engage mid‐to late‐career staff.

**Implications for Nursing Practice:**

The findings support investment in transformational leadership development, direct support for clinical performance, a stronger feedback culture, continuing professional development—particularly for mid‐ to late‐career nurses—and quality‐improvement efforts within the Health Cluster system. *No patient or public contribution:* While the study acknowledges the vital participation of nursing staff at the Tabuk Health Cluster, patients and members of the public were not involved in the design, conduct or reporting of this research.

## 1 Introduction

Nurses are central to any healthcare system, serving as the front‐line link between clinical policy and patient outcomes (AL‐Dossary 2018). Improving their clinical performance is not only an operational goal but a strategic requirement for high‐quality care (Yuxiu et al. 2011). In Saudi Arabia, this role has been elevated under Saudi Vision 2030, which aims to develop a more integrated, efficient and patient‐centred healthcare system. Central to this is the professionalization of nursing to meet international benchmarks (AL‐Dossary 2018).

Leadership style is an important correlate of organizational climate and staff efficiency in nursing management (Specchia et al. 2021). Transformational approaches in particular have been linked with greater empowerment, psychological safety and clinical innovation among nurses (Ye and Liu 2022). As healthcare organizations become more complex, the leadership style adopted by nursing managers is relevant to clinical performance, particularly during systemic change (Renkema et al. 2021; Shakil et al. 2023).

Currently, the Saudi Ministry of Health is implementing a Health Cluster Transformation model. By grouping hospitals and primary care centres into integrated “Clusters,” such as the Tabuk Health Cluster, the system aims to streamline care delivery. This organizational shift requires nursing leadership that can support the transition while maintaining high standards of care (AL‐Dossary 2018). Understanding how current leadership practices relate to frontline performance within these clusters is relevant to the national health reforms.

Despite the established link between leadership and work outcomes in global literature, these dynamics have rarely been examined within Saudi Arabia's new integrated clusters. While previous studies have explored nursing performance generally (Song et al. 2017), few have analysed how the health‐cluster environment and varying staff experience relate to this association (Tvedt et al. 2012).

Internationally, the association between nursing leadership and clinical outcomes is well established. A systematic review by (Sfantou et al. 2017) reported that leadership style was strongly associated with quality‐of‐care measures and was a core component of integrated care. Examine nurse Outcomes, (Wong et al. 2013) found that relational and transformational leadership were associated with higher patient satisfaction and lower rates of mortality, medication errors and hospital‐acquired infections. Consistent with this, an updated review found relationship‐oriented leadership positively associated with better workforce and work environment outcomes, whereas task‐oriented styles were negatively or inconsistently related (Cummings et al. 2018). Single site and multi‐country studies support this pattern: communication competences predicted nursing performance in intensive‐care nurses in South Korea (Song et al. 2017), and leadership style was associated with job satisfaction across Europe (Specchia et al. 2021). Comparatively little is known, however, about how leadership relates to clinical performance in the Gulf region, and specifically within Saudi Arabia's newly integrated clusters—the gap addressed by this study.

Building on this gap, the present study examines the association between transformational leadership and the clinical performance of nurses within the Tabuk Health Cluster. By analysing the correlation between leadership behaviours and self‐reported performance, it aims to identify performance‐related factors that could inform targeted leadership development under the Vision 2030 health reforms.

## 2 Methodology

### 2.1 Design

A cross‐sectional quantitative design was used, with data collection through a self‐administered online questionnaire over an approximately six weeks period (November 2024 to January 2025).

### 2.2 Setting

This study was conducted in the Tabuk Health Cluster of Saudi Arabia.

### 2.3 Population and Sample

#### 2.3.1 Sampling

Sample size was estimated using Cochran's (1977) formula. For a large population, the formula indicated a minimum of 384:

$$\;n_{0}=z^{2}\cdot p\cdot \left(1-p\right)/e^{2}={\left(1.96\right)}^{2}\times 0.5\times 0.5/{\left(0.05\right)}^{2}=384.16$$

where z = 1.96 (95% confidence level), *p* = 0.5 (expected proportion, set at 0.5 to maximize the required sample), and e = 0.05 (margin of error). As the exact number of nurses in the Tabuk Health Cluster was not publicly available, the population was approximated from Ministry of Health workforce statistics. The sample achieved, recruited by convenience sampling, was 280—below this large‐population estimate. Applying a finite‐population correction for an estimated population of approximately 3000 nurses, a sample of 280 corresponds to an approximate margin of error of 5.6% at the 95% confidence level. This shortfall and the use of convenience sampling are acknowledged as limitations and should be considered when interpreting the findings.

#### 2.3.2 Population

*Inclusion Criteria*

- Employment as a nurse or nurse leader in the Tabuk Health Cluster of Saudi Arabia.
- Any sex, nationality and age
- At least 1 year of work experience as a nurse or nurse leader

*Exclusion Criteria*

- Refusal to participate in the study.
- Extended leave or vacation during the study period
- Incomplete or missing study data

### 2.4 Measurement Tool

A demographic information form was used to evaluate the characteristics of participants including age, sex, educational level, marital status, current occupational position and employment status. These data were gathered to obtain a comprehensive understanding of the background and demographics of participants.

Transformational leadership was measured using the Global Transformational Leadership (GTL) scale (Carless et al. 2000), a seven‐item measure of transformational leadership behaviours, each rated on a five‐point Likert scale. Clinical performance was assessed using a 51‐item scale adapted from (Yuxiu et al. 2011), which evaluates nursing work performance across multiple dimensions; each item is scored on a 4‐point Likert scale (1–4), with higher scores indicating better performance.

Both instruments were made publicly available for non‐commercial academic research without any separate permission from the original developers, as long as citations for the original sources are included. References to the original development and validation studies of both instruments were made appropriately.

#### 2.4.1 Study Variables

The dependent variable of the study was Clinical Performance, while the independent variable was transformational leadership.

#### 2.4.2 Data Validity and Reliability

The level of enthusiasm for a subject can be gauged using a Likert scale, which is widely employed in surveys. This scale is based on a middle ground that shows how strongly people feel about various issues (McLeod et al. 2012).

Table 1 shows that the Pearson correlation coefficients between each item and the transformational leadership scale were all significant at the 0.01 level. These positive coefficients indicated a high level of internal consistency. The strong correlation between the individual items and the overall scale demonstrated the scale's validity.

**TABLE 1:** Internal consistency of the transformational leadership scale.

Item no.	Pearson correlation coefficient	Item no.	Pearson correlation coefficient
1	0.799^a^	5	0.866^a^
2	0.883^a^	6	0.849^a^
3	0.860^a^	7	0.810^a^
4	0.886^a^		

^a^ Significant at *α* ≤ 0.01.

As presented in (Table 2.), the Pearson correlation coefficients between each item and the overall clinical performance scale were significant at the 0.01 level. These positive coefficients showed a strong internal consistency, demonstrating the validity of the scale.

**TABLE 2:** Internal consistency of the Clinical Performance Scale.

Item no.	Pearson correlation coefficient	Item no.	Pearson correlation coefficient
1	0.441^a^	27	0.572^a^
2	0.528^a^	28	0.542^a^
3	0.503^a^	29	0.564^a^
4	0.513^a^	30	0.497^a^
5	0.520^a^	31	0.559^a^
6	0.453^a^	32	0.478^a^
7	0.545^a^	33	0.536^a^
8	0.533^a^	34	0.561^a^
9	0.462^a^	35	0.554^a^
10	0.516^a^	36	0.509^a^
11	0.549^a^	37	0.527^a^
12	0.588^a^	38	0.458^a^
13	0.513^a^	39	0.560^a^
14	0.566^a^	40	0.566^a^
15	0.513^a^	41	0.548^a^
16	0.473^a^	42	0.461^a^
17	0.539^a^	43	0.521^a^
18	0.556^a^	44	0.521^a^
19	0.569^a^	45	0.494^a^
20	0.480^a^	46	0.510^a^
21	0.528^a^	47	0.483^a^
22	0.497^a^	48	0.504^a^
23	0.503^a^	49	0.577^a^
24	0.492^a^	50	0.569^a^
25	0.532^a^	51	0.329^a^
26	0.509^a^		

^a^ Significant at *α* ≤ 0.01.

As illustrated in Table 3. Cronbach's alpha coefficient was 0.936 for the transformational leadership scale and 0.945 for the Clinical Performance scale, indicating that the study instruments were highly reliable.

**TABLE 3:** Cronbach's alpha coefficients of the study instruments.

No.	Scale	No. of items	Cronbach's alpha coefficient
1	Transformational leadership scale	7	0.936
2	Clinical Performance scale	51	0.945

### 2.5 Data Collection

First IRB approval obtained from the University of Tabuk, Local Research Ethics committee (LREC) (Approval no: UT‐463‐261‐2024; Date approved: 21 November 2024). In the first page of the online questionnaire, all participants were informed by the researcher that this study was composed of two parts and was to be carried out with total confidentiality and respect for their privacy. Participants were free to identify themselves in any way they wanted, and their information would not be exposed. After questionnaire distribution, all data were treated, analysed and presented as group data in Excel sheets. Suitable statistical tests were used for data analysis.

### 2.6 Data Analysis

Data were analysed using SPSS version 26. Descriptive statistics, specifically frequencies, percentages, means and standard deviations, were utilized. In addition, Cronbach's alpha coefficients were calculated to quantify the correlation and coherence between the questionnaire items, determine the consistency in measuring the concept and estimate the quality of the measures. The independent samples *t*‐test and ANOVA were also conducted. Prior to regression, the assumptions of linearity, normality of residuals, homoscedasticity and absence of influential outliers were examined; effect sizes (*η* ^2^) were calculated for ANOVA.

## 3 Results

Table 4 describes the demographic profile of 280 nursing professionals. A large proportion (42.5%) were aged 36‐49 years, and just over half (52.9%) held bachelor's degree. Nearly half (47.9%) had 1–5 years of experience in critical care units. The sample was therefore predominantly early‐to‐mid career.

**TABLE 4:** Demographic characteristics of participants (*N* = 280).

Variable	Category	*n*	%
Age	< 25 years	50	17.9
	25–35 years	71	25.4
	36–49 years	119	42.5
	> 49 years	40	14.3
Sex	Male	148	52.9
	Female	132	47.1
Nursing qualification	Diploma	132	47.1
	Bachelor's degree	148	52.9
Job position	Head nurse	106	37.9
	Staff nurse	174	62.1
Years of experience in critical care units	< 1 year	51	18.2
	1–5 years	134	47.9
	> 5 years	95	33.9

As shown in Table 5, nurses reported a high level of perceived leadership within their units, with an overall mean score of 3.96. This indicates a favourable baseline for leadership‐focused interventions consistent with Vision 2030 workforce goals.

**TABLE 5:** The perceived leadership levels among nursing staff.

No.	Item	Mean	SD
1	Communicates a clear and positive vision of the future	3.97	1.28
2	Treats staff as individuals and supports and encourages their development	3.97	1.22
3	Provides encouragement and recognition to staff	3.97	1.21
4	Fosters trust, involvement and cooperation among team members	3.96	1.22
5	Encourages innovative thinking by approaching problems from new perspectives and questions assumptions	3.91	1.24
6	Is clear about his/her values and practices what he/she preaches	3.93	1.19
7	Instils pride and respect in others and inspires staff by being highly competent	4.04	1.14
	Total scale score	3.96	1.03

*Note:* Leadership was measured using the Global Transformational Leadership (GTL) scale (Carless et al. 2000).

As shown in Tables 6 and 7, Clinical Performance was moderate in the leadership (mean = 2.75) and critical care (mean = 2.79) domains. These findings suggest opportunities for professional development, particularly in aligning frontline practice within international quality standards.

**TABLE 6:** Clinical work performance in leadership subscale scores.

No.	Item	Mean	SD
1	Teach patients' family members about patients' needs.	2.59	0.90
2	Coordinate the nursing care plan with the medical care plan.	2.66	0.85
3	Provide praise and recognition for achievement to those under their direction.	2.73	0.86
4	Teach preventive health measures to patients and their families.	2.78	0.87
5	Identify and use community resources in developing a plan of care for patients and their families.	2.71	0.85
6	Identify and include anticipated changes in patients' conditions in the nursing care plan.	2.78	0.84
7	Evaluate the results of nursing care.	2.83	0.87
8	Promote the inclusion of patients' decisions and desires concerning their care.	2.78	0.90
9	Develop nursing care plans for patients.	2.83	0.89
10	Initiate planning and evaluation of nursing care with others.	2.78	0.87
	Total subscale score	2.75	0.56

*Note:* Clinical Performance measured using the 51‐item scale adapted from (Yuxiu et al. 2011).

**TABLE 7:** Clinical work performance in critical care subscale scores.

No.	Item	Mean	SD
1	Perform technical procedures (e.g., oral suctioning, tracheostomy care, IV therapy, catheter care and dressing changes).	2.76	0.92
2	Adapt teaching methods and materials to the understanding of the particular audience (e.g., patients' age, educational background and sensory deprivation).	2.70	0.88
3	Identify and include immediate patient needs in the nursing care plan.	2.81	0.84
4	Develop innovative methods and materials for teaching patients.	2.86	0.85
5	Communicate a feeling of acceptance of each patient and a concern for the patient's welfare.	2.77	0.84
6	Seek assistance when necessary.	2.87	0.88
7	Help patients communicate with others.	2.76	0.88
8	Use mechanical devices (e.g., suction machine, Gomco, cardiac monitor and respirator).	2.80	0.85
	Total subscale score	2.79	0.61

*Note:* Clinical Performance measured using the 51‐item scale adapted from (Yuxiu et al. 2011).

Table 8, shows moderate Clinical Performance across domains (leadership Mean = 2.75 and critical care Mean = 2.79), reflecting a moderate level of competency that targeted institutional support could help improve towards international standards.

**TABLE 8:** Clinical work performance scale scores.

No.	Subscale	Mean	SD
1	Leadership	2.75	0.56
2	Critical care	2.79	0.61
3	Teaching/collaboration	2.67	0.56
4	Planning/evaluation	2.67	0.56
5	Interpersonal relationships/communication	2.68	0.59
6	Professional development	2.66	0.56
	Total scale score	2.70	0.45

As shown in Tables 9 and 10, a significant disparity was observed based on years of experience. Nurses with 1–5 years of experience demonstrated higher levels of both leadership perception (*F* = 15.16, *p* < 0.001) and Clinical Performance (*F* = 8.29, *p* < 0.001). For the differences by years of experience, effect sizes were *η* ^2^ = 0.10 for leadership perception and *η* ^2^ = 0.06 for Clinical Performance, indicating medium effects.

**TABLE 9:** Difference in the level of leadership according to the demographic characteristics.

Variable		Mean	Test	Test statistic	*p*
Age	< 25 years	3.86	ANOVA	*F* = 0.828	0.479
	25–35 years	4.09			
	36–49 years	3.99			
	> 49 years	3.81			
Sex	Male	4.06	Independent samples *t*‐test	*t* = 1.171	0.088
	Female	3.85			
Nursing qualification	Diploma	4.05	Independent samples *t*‐test	*t* = 1.277	0.202
	Bachelor's degree	3.89			
Job position	Head nurse	4.08	Independent samples *t*‐test	*t* = 1.461	0.145
	Staff nurse	3.89			
Years of experience in critical care units	< 1 year	4.30^a^	ANOVA	*F* = 15.16	< 0.001*
	1–5 years	4.15^a^			
	> 5 years	3.52^b^			

*Note:* *Significant at *p* < 0.05. The test statistic is F for ANOVA (Age, Years of experience) and t for the independent‐samples *t*‐tests (Sex, Nursing qualification, Job position). ^a,b^Means not sharing a common superscript letter differ significantly (Dunnett's C post hoc test, *p* < 0.05).

**TABLE 10:** Difference in the level of clinical work performance according to the demographic characteristics.

Variable		Mean	Test	Test statistic	*p*
Age	< 25 years	2.65	ANOVA	*F* = 0.326	0.807
	25–35 years	2.70			
	36–49 years	2.72			
	> 49 years	2.72			
Sex	Male	2.72	Independent samples *t*‐test	*t* = 0.729	0.467
	Female	2.68			
Nursing qualification	Diploma	2.74	Independent samples *t*‐test	*t* = 1.227	0.221
	Bachelor's degree	2.67			
Job position	Head nurse	2.70	Independent samples *t*‐test	*t* = −0.114	0.909
	Staff nurse	2.71			
Years of experience in critical care units	< 1 year	2.87^a^	ANOVA	*F* = 8.29	< 0.001*
	1–5 years	2.74^a^			
	> 5 years	2.57^b^			

*Note:* *Significant at *p* < 0.05. The test statistic is F for ANOVA (Age, Years of experience) and t for the independent‐samples *t*‐tests (Sex, Nursing qualification, Job position). ^a,b^Means not sharing a common superscript letter differ significantly (Dunnett's C post hoc test, *p* < 0.05).

### 3.1 Association Between Transformational Leadership and Clinical Performance

Regression assumptions were examined. The leadership–performance relationship was linear (Pearson *r* = 0.52, *p* < 0.001). Residuals were approximately normal (skewness = −0.59, kurtosis = 2.47), although the Shapiro–Wilk test was significant (W = 0.96, *p* < 0.001), as is common in large samples. The Breusch–Pagan test indicated heteroscedasticity (LM = 9.95, *p* = 0.002), so heteroscedasticity‐consistent (HC3) standard errors were reported, and the predictor remained significant. No influential outliers were found: three cases exceeded a studentized residual of ±3, but all Cook's distances were below 1 (maximum = 0.14).

As shown in Table 11, transformational leadership was significantly associated with clinical performance, explaining 26.9% of the variance (*R* ^2^ = 0.269, *F* = 103.875, *p* < 0.001). These results indicate that transformational leadership is a significant, modifiable predictor of Clinical Performance.

**TABLE 11:** Regression analysis of the relationship between transformational leadership and clinical performance.

*R*	*R* ^2^	*F*	*p*	*β*	*t*	*p*
0.522	0.269	103.875	< 0.001	0.522	10.192	< 0.001

*Note:* Statistical significance determined at *p* < 0.05.

## 4 Discussion

This study examined the relationship between nursing leadership and clinical performance in the context of Saudi Arabia's Vision 2030 healthcare goals (Alilyyani et al. 2022). As the Kingdom implements its Health Cluster Transformation towards more integrated care models, nursing leadership is one relevant factor for clinical performance and organizational efficiency (AL‐Dossary 2018). The high perception of leadership in this study (Mean = 3.96) is consistent with the national emphasis on developing the healthcare workforce (El Muksoud and Metwally 2022); however, the moderate clinical performance observed suggests that leadership perceptions were not fully reflected in frontline performance. The significant association between transformational leadership and performance indicates that leadership is one factor relevant to the new healthcare delivery model within the Tabuk Health Cluster.

Among the 280 participants, the majority (42.5%) were aged 36–49 years, consistent with the age range reported in several previous studies (Al‐Haroon and Al‐Qahtani 2020; Almazan et al. 2019; Ibrahim et al. 2016). The sex distribution was nearly equal, with the male and female participants comprising 52.9% and 47.1% of the sample, respectively. In contrast, Alsadaan et al. (2021) and Alharbi et al. (2019) reported that most registered nurses in Saudi Arabia were women.

Approximately 62.1% of the participants in this study were staff nurses, while 37.9% were head nurses. This diversity in nursing positions indicates a varied sample of nursing professionals. Conversely, Alluhidan et al. (2020) emphasized the importance for nursing staff to pursue further education after graduation to raise their quality of work and care. Regarding experience, most of the participants (47.9%) had worked for 1–5 years in critical care units. This degree of expertise helped support the research objectives and achieve the intended goals.

In this study, the participants demonstrated a high level of leadership, with a mean score of 3.96, which agrees with the report by El Muksoud and Metwally (2022). The authors found that 75% of staff nurses possessed a positive perception of leadership, and 37% of nurse leaders exhibited a significant level of leadership. The same finding was reported by Ali et al. (2020) where 58.8% of head nurses showed a high degree of leadership behaviours and Alharbi et al., 201939.9% were at a moderate level. However, the multi‐centre cross‐sectional study (Ofei et al. 2023) revealed that an average leadership score of 3.71 was obtained by 76.0% of nurses.

The level of clinical work performance in leadership among the participants in the current study was moderate, with a mean score of 2.75. This finding aligns with the report by Khalaaf et al. (2022), showing that 67.6% of staff nurses exhibited a moderate level of clinical work performance, while 56.4% had a high level of clinical work performance in terms of leadership. Gunawan et al. (2019) similarly reported that the level of performance of nurses was moderate and influenced by several aspects, including perfect leadership. Conversely, the present study showed a moderate level of clinical work performance in critical care, with a mean score of 2.79. In contrast, Ahmed et al. (2018) found that 63% of staff nurses exhibited a high level of clinical work performance in critical and intensive care units.

The analysis of clinical work performance in teaching and collaboration revealed a moderate level of performance among participants, with the highest mean score observed for the item ‘Guide other health team members in planning for nursing care’ (Mean = 2.78). Conversely, the lowest score was recorded for ‘Provide emotional support to the families of dying patients’ (Mean = 2.45). The findings suggest nurses are capable of orienting peers but less adept at providing emotional support, an essential part of a supportive environment for patients and families. It was also observed (Al‐Ajarmeh et al. 2022) to have a significant correlation between teaching and job performance of 155 critical care nurses. Among the subscales of nurse–nurse collaboration, only conflict management emerged as an independent significant predictor of self‐assessed nursing clinical work performance (*p* = 0.003).

Regarding clinical work performance in planning and evaluation, participants exhibited a moderate level of performance, with the highest mean score for the item ‘Contribute to the nursing care plan for patients’ (Mean = 2.81). On the contrary, ‘Promote the use of interdisciplinary resource persons’ scored lowest (Mean = 2.48). This shows that nurses are in fact engaged in the development of care plans, but there is a need for further integration with interdisciplinary teams to bolster patient outcomes. For clinical work performance based on interpersonal relationships and communication, the mean for item ‘Function calmly and competently in emergency situations’ was high (Mean = 2.83), the analysis indicating an overall moderate level of performance on this subscale. But the lowest scores were noted for ‘Recognize and meet the emotional needs of dying patients’ and ‘Communicate facts, ideas and professional opinions in writing to patients and their families’ (Mean = 2.56). These results indicated that improving communication skills of nurses to take care of the emotional needs of patients is an integral part of holistic care. This accords with the findings of (Song et al. 2017). The authors conducted a cross‐sectional study among 197 intensive care unit staff nurses across three tertiary academic medical centres, revealing that professional communication competencies were the sole significant predictors of nursing performance after controlling for sociodemographic factors. The enhanced professional communication competencies of nurses correlated with increased age, higher educational attainment, extended years of clinical and intensive care unit experience and higher monthly compensation.

Regarding the professional development of the participants, they performed moderately well, with the highest mean score for the item ‘Demonstrate self‐confidence’ (Mean = 2.86). On the other hand, ‘accept and use constructive criticism’ got the lowest score (Mean = 2.14). What this suggests is that nurses, overall, appear to be confident in themselves, but there is still considerable room for improvement when it comes to a culture in which constructive feedback and continuous professional development are fostered. Similarly, (Osei et al. 2019) reported that nursing administrators showed considerable professional development in recommending and supporting nursing scientific performance and management.

There was a significant difference in transformational leadership according to the years of experience in critical care units (F = 15.16, *p* < 0.001), with participants with > 5 years of experience showing a lower level of leadership than their counterparts. This finding aligns with the report by (den Breejen‐de Hooge et al. 2021), wherein sex, occupation and practice area were noted as major determinants of nursing leadership. (Tvedt et al. 2012) also demonstrated that process measures and experiences were associated with nurse‐reported leadership. No connections were seen between nursing engagement, education, career and ward leadership. In the present study, the clinical performance of the participants also significantly differed according to the years of experience in critical care units (*F* = 8.29, *p* < 0.001), with the participants with > 5 years of experience demonstrating a lower level of clinical performance than their counterparts. These results match those of (Al‐Hamdan et al. 2017) but contrast those of (Delak and Širok 2022), who reported significant differences in nursing clinical performance according to sex, years of experience and educational background.

A key finding of this study is the predictive value of transformational leadership for clinical performance. The regression model indicated that leadership explained 26.9% of the variance in performance. Because leadership, unlike demographic factors, is modifiable, developing transformational leadership among nursing managers may be associated with improvements in clinical performance and patient safety, although the cross‐sectional design does not permit causal conclusions. (Priyantini and Ayatulloh 2023) reported similar findings, with 65.7% of nurses showing high levels of nursing leadership and clinical performance and a significant association between them (*R* = 0.728, *p* < 0.001). (Gurusinga et al. 2023) also found that the clinical performance of nurses strongly correlated with their leadership (*p* = 0.015). Similarly, (As et al. 2023) reported that 65.8% of nurses demonstrated high levels of job performance and that job performance was significantly associated with leadership (*p* = 0.000). Further studies can also control potential confounding factors (e.g., organization culture and external context that could influence nurse behaviour) to better understand the relationship between transformational leadership and clinical work performance.

### 4.1 Limitations

- The cross‐sectional nature of the study limits the possibility of assessing causality. Future research should explore the temporal dynamics of transformational leadership through longitudinal studies.
- We used convenience sampling for this study that can cause sample selection bias; therefore, the results might be restricted to generalization into the wider Saudi Arabia nursing population.
- The study was small compared to the target population of nurses in the Tabuk region. Nursing work performance in terms of care quality and patient outcomes from the perspectives of patients was not studied.
- Clinical performance was self‐reported, which may introduce social‐desirability and common‐method bias and tends to inflate ratings. The shared method variance is one possible explanation for the relationship observed because leadership and performance were rated by the same respondents at a single point in time. Future research should involve supervisor/peer ratings or some indicators of performance other than self‐evaluation.

## 5 Conclusion

Transformational leadership among nurses in the Tabuk Health Cluster significantly predicted clinical performance and highlights the role of developing leaders as part of the cluster transition and new national health priorities (Vision 2030). In an unexpected result, nurses working for more than 5 years reported lower leadership and performance scores compared to their less experienced counterparts. The lack of in‐house programmatic steps to support professional development suggests that these initiatives need not be focused solely on early‐career staff; mid‐ to late‐career nurses need to be re‐engaged through tailored and supportive programmes as well. Strengthening leadership skills in this group may help reduce the observed performance gap and sustain the quality gains sought by the current health reforms.

## 6 Recommendations

To advance the goals of the Saudi Vision 2030 and build upon the findings of this study, the following is recommended:

- National policy: The Ministry of Health could incorporate transformational leadership competencies into the national nursing framework so that leadership development is applied consistently across health clusters
- Longitudinal tracking: Future studies should use longitudinal designs to follow how clinical performance changes as the Tabuk Health Cluster reaches full operation.
- Expanded scope: Further research should include qualitative interviews with senior nurses to explore the reasons behind their lower leadership and performance scores.

## 7 Implications for Nursing Practice

The findings of this study offer several strategic applications for nursing administrators within the Saudi Health Cluster system:

- Support for senior nurses: Managers should provide targeted professional development for nurses with more than 5 years' experience to address the lower performance observed in this group.
- Leadership in appraisals: Because leadership accounted for about 27% of the variance in performance, leadership behaviours could be included in formal nursing performance appraisals.
- Use of subscale data: The performance scale includes a subscale structure that allows managers to diagnose specific areas, such as critical care, where targeted interventions are more likely to yield better results.
- Feedback culture: The low score on accepting constructive criticism suggests a need to build a supportive feedback culture within nursing teams.
- Quality improvement: Embedding leadership and performance data into routine quality‐improvement cycles can help translate findings into measurable practice change.
- Vision 2030 Alignment: Nurse leaders can operationalize priorities of Vision 2030 and implement them in daily clinical practice to shift performance from a moderate level towards a higher level.

## Author Contributions

All authors of this study have participated equally.

## Funding

The authors have nothing to report.

## Ethics Statement

Ethical approval was obtained from the Institutional Review Board (IRB) in Tabuk University (*Approval No: UT‐463‐261‐2024*). Furthermore, participants were given clear information and asked for their consent to participate in the study voluntarily.

## Consent

Informed consent was obtained from all participants involved in the study.

## Conflicts of Interest

The authors declare no conflicts of interest.

## Data Availability

The datasets generated and analysed during this study are available from the corresponding author.
